# Percutaneous Mitral Valvotomy in a Case of Situs Inversus Totalis and Juvenile Rheumatic Critical Mitral Stenosis: Case Report

**DOI:** 10.14740/jocmr2473w

**Published:** 2016-02-27

**Authors:** Santosh Kumar Sinha, Ramesh Thakur, Mukesh Jitendra Jha, Karandeep Singh Sayal, Mohit Sachan, Vinay Krishna, Ashutosh Kumar, Vikas Mishra, Chandra Mohan Varma

**Affiliations:** a Department of Cardiology, LPS Institute of Cardiology, G.S.V.M. Medical College, Kanpur, Uttar Pradesh 208002, India

**Keywords:** Dextrocardia, Juvenile rheumatic mitral stenosis, Inoue technique, Percutaneous mitral valvotomy, Situs inversus totalis

## Abstract

Situs inversus totalis is a rare congenital disorder where the heart being a mirror image is situated on the right side of the body. Distorted cardiac anatomy makes fluoroscopy-guided percutaneous mitral valvotomy (PMV) technically challenging and there are only few reports of PMV in situs inversus totalis. Here we report a case where PMV was successfully done for situs inversus totalis with rare coincidence of juvenile rheumatic severe mitral stenosis in a 12-year-old boy with a few modifications of standard Inoue technique. He had exertional dyspnea of NYHA class III with initial mitral valve area (MVA) of 0.6 cm^2^ and severe pulmonary arterial hypertension with features suitable for PMV. Femoral vein was accessed from the left side to align the septal puncture needle and balloon to facilitate left ventricular entry. Septal descent and puncture by Brockenbrough needle was performed in the right anterior oblique view with the needle facing 5 o’clock position. Accura balloon was negotiated across mitral valve in left anterior oblique and procedure was successfully executed. Echocardiography showed a well-divided anterior commissure with an MVA of 2.0 cm^2^ and mild mitral regurgitation. In summary, PMV is safe and feasible in the rare patient with situs inversus totalis with few modifications of the Inoue technique.

## Introduction

Situs inversus totalis refers to the heart being a mirror image situated on the right side with all visceral organs to be mirrored with incidence of approximately 1 in 12,000 people [1], while one in three of these will have situs inversus. Distorted cardiac anatomy makes fluoroscopy \guided transcatheter procedures difficult which become technically more challenging in the cases with percutaneous mitral valvotomy (PMV), where the cardiac malpositions substantially increase the complications beginning from interatrial septal puncture to left ventricular entry. There are only few reports on successful percutaneous transvenous mitral commissurotomy (PTMC) in abnormal cardiac anatomy using the standard Inoue technique [2-7]. Here we describe a case of a 12-year-boy with situs inversus totalis, where PTMC was successfully performed with slight modifications of the standard Inoue technique. Mitral valvuloplasty is treatment of choice to correct severe mitral stenosis in a selected subset of patients.

## Case Report

**Figure 1:**
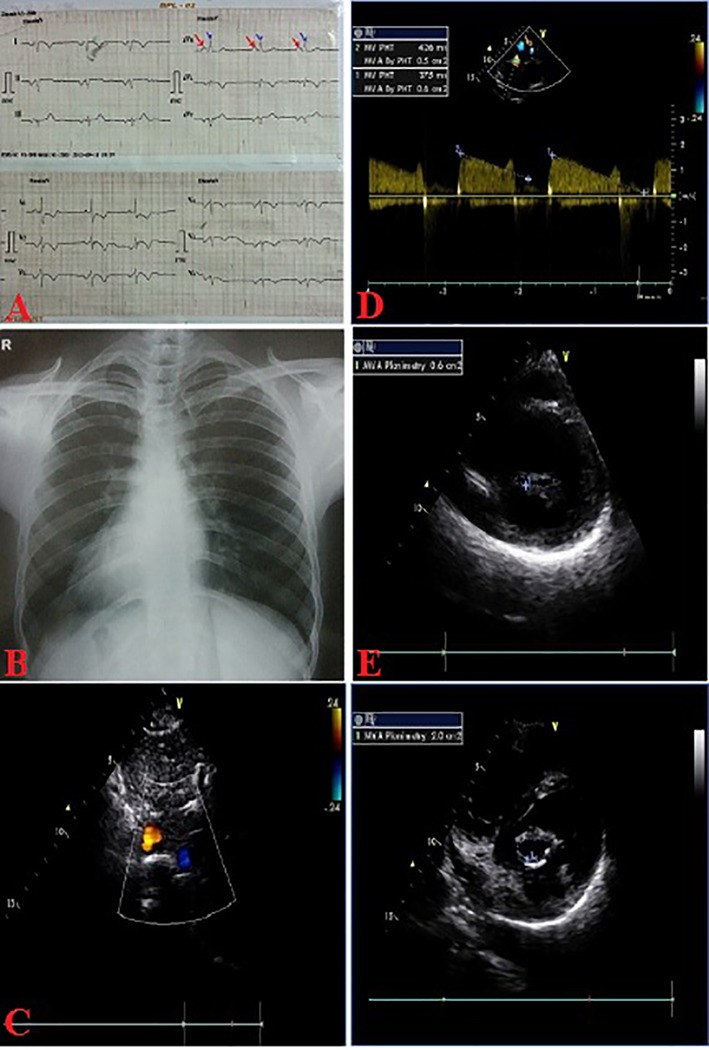
ECG (A); chest X-ray (B) PA view showing situs inversus totalis; subcostal echo showing situs inversus (C), critical MS by PHT (D), planimetry (E), post-PTMC MVA-2.0 cm^2^ (F).

**Figure 2:**
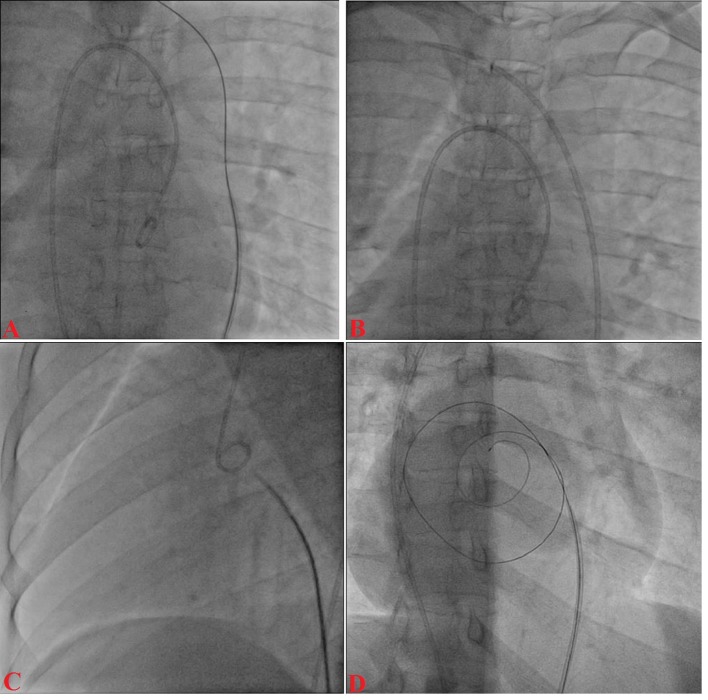
J-tip guide wire into the left-sided superior vena cava (A), 8F Mullins sheath into the left SVC (B); Brockenbrough needle oriented to 9 o’clock position (C); septal puncture (D).

**Figure 3:**
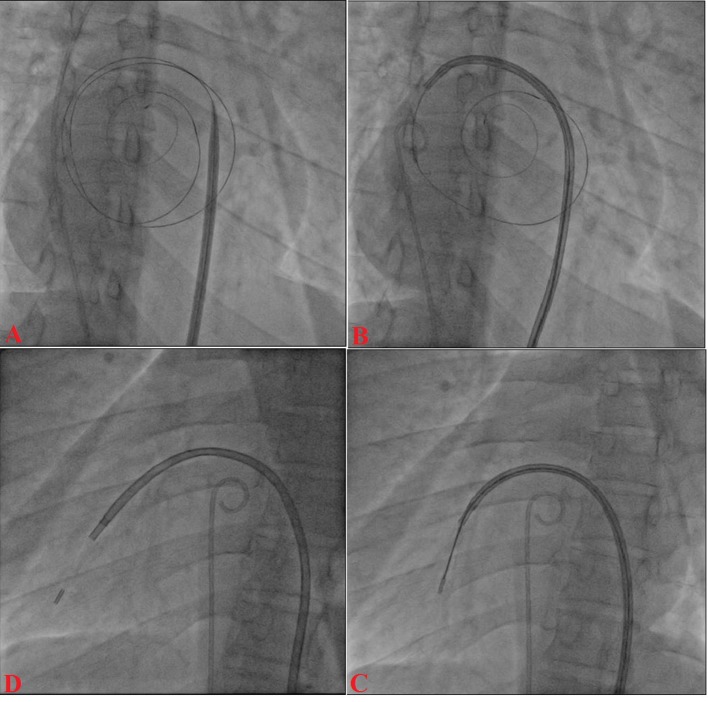
14F dilator across the septum (A); accura balloon into LA over looped wire (B); director inside the balloon (C); balloon towards mitral valve (D).

**Figure 4:**
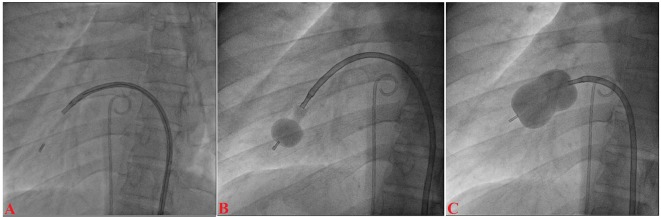
Accura balloon entering into the LV (A); distal inflation of balloon (B); mitral valve dilation (C).

A 12-year-old boy presented with progressive exertional dyspnea NYHA class III of 7 months duration. Blood pressure was 100/76 mm Hg in right arm in supine position. Pulse rate was 70/min, regular, low volume, with no special character with all peripheral pulses palpable. Jugular venous pulse pressure was elevated 6 cm above sternal angle with prominent a wave and v-y descent. Apex beat was located in right fifth intercostals space, tapping in character. There was grade II right parasternal heave. P_2_ was palpable. S_1_ was loud. S_2_ was loud; loud P_2_ component with narrow split. Opening snap (OS) was present with narrow A_2_-OS gap. There was long grade IV mid diastolic rumbling murmur with presystolic accentuation. Pansystolic murmur grade III/VI of tricuspid regurgitation was present in left parasternal area. The electrocardiograph (ECG) showed inverted P wave in all leads except aVR and V_1_, upright R wave in aVR, reduction in the R wave voltage across the chest leads with evidence of right ventricular hypertrophy (Fig. 1A). Chest X-ray PA view showed dextrocardia (Fig. 1B). Subcotal window showed aorta on the right and inferior vena cava on the left side, i.e. situs inversus (Fig. 1C). Transthoracic echocardiography showed critical mitral stenosis with mitral valve area (MVA) of 0.6 cm^2^ by pressure half time (Fig. 1D) and planimetry (Fig. 1E) and mild mitral regurgitation. His Wilkin’s score was 8/16 (C_2_, T_2_, M_2_, S_2_). Mean gradient across mitral valve was 36 mm Hg. Transesophageal echo ruled out any LA or LAA clot. Dextrocardia, atrio-ventricular (AV) and ventriculo-arterial (VA) concordance were present. Left femoral artery and venous access was obtained with a 5F arterial and 8F venous sheaths, respectively after proper consent. A 5F pigtail catheter was passed retrograde into the aorta and parked in the non-coronary sinus of the aortic root. A 0.035" J-tip guide wire was then passed up the femoral vein into the inferior vena cava (IVC) and up into the left-sided superior vena cava (SVC) via the left-sided “right atrium” (Fig. 2A). An 8F Mullins sheath was passed up on the guide wire, into the left SVC (Fig. 2B). A curved Brockenbrough septal puncture needle was introduced into the sheath stopping just short of the tip and was oriented to 9 o’clock position in the SVC. Septal descent was done by withdrawing the needle and the sheath in tandem into the heart with the needle pointer in 9 o’clock position. Septal puncture was done in right anterior oblique (RAO) 40° and also confirmed in left lateral projection which was contrary to our conventional view, i.e., left anterior oblique (LAO) 40° projection (Fig. 2C). LA was entered and pressure was recorded. The septum was dilated and heparin 100 IU/kg IV was given. A “loopy” LA wire was then passed through the sheath into LA and the latter was withdrawn leaving the LA wire inside (Fig. 2D). The 14F dilator was then used to dilate the septum (Fig. 3A). Subsequently, the accura balloon (Vascular Concepts, Essex, UK) was introduced over the LA wire to enter the LA (Fig. 3B). The LA wire was withdrawn and the balloon was flushed and simultaneous LA/LV pressures were recorded. Now, director was used to guide the balloon (Fig. 3C) towards mitral valve by slight counter clock rotation till flip at the balloon tip was noted (Fig. 3D). Director was pulled and balloon was pushed in a symphony to facilitate its entry into the LV (Fig. 4A). Mitral valve was negotiated and dilation was performed successfully in LAO 40° projection which was contrary to our conventional view, i.e., RAO 40° projection (Fig. 4B, C). Post-procedural MVA was 2.0 cm^2^ and MR still remained mild (Fig. 1F). Patient was discharged in stable condition on third day.

## Discussion

Mirror-image dextrocardia, as in our case, has been estimated to occur with a prevalence of 1:10,000 patients. However, there are only a few case reports in the literature on PTMC in similar settings [5]. This might be due to the fact that many of these patients undergo surgical commissurotomy due to the technical difficulties involved in a percutaneous procedure in general. Trans-septal catheterization is considered a technical challenge in anatomically malpositioned hearts, as it is fraught with a higher risk of cardiac perforation. It is performed from the left groin as it reduces the puncture needle angulation at the confluence of the iliac veins to the left-sided IVC thus helping to align the tracking of balloon and septal puncture needle with IVC and common iliac vein. The descent of the needle assembly is recommended in mirror-image position, i.e. 7 - 9 o’clock position of the external indicator of the needle instead of its usual 4 - 6 o’clock position [8]. After septal puncture, entry into the LA and its depth can be confirmed by pressure measurement and contrast staining of the left atrium [5, 6, 8]. Septal dilatation is to be done in usual fashion. Accura balloon is mounted over the LA wire. After removing the looped LA wire, director should be entered into accrue balloon and negotiation across the mitral valve should be done in RAO view. Director should be rotated in counter clock fashion. Once the flip at the tip of balloon is observed, director should be removed as it facilitates entry into LV. Thereafter further steps remain the same. Transesophageal and intracardiac echo may be important adjunctive sometimes but also cumbersome otherwise [9]. The trans-jugular approach is thought to overcome many of the technical problems encountered with the transfemoral route in cases with anatomical alterations [10]. Furthermore, the radiographic images can be acquired in the inverted position and can be used as fluoroscopic guidance for septal puncture but this may not be available in many cathlab equipments [5]. All technical modifications have been summarized in Table 1. Despite the challenging anatomy, PTMC has been demonstrated to be a safe and feasible option for MS in patients with unusual cardiac anatomy [10].

**Table 1:** Summary of Technical Modifications for PTMC in Dextrocardia

Steps	Conventional PTMC	PTMC in dextrocardia
Trans-septal catheterization	Right groin	Left groin
Descent of needle assembly	4 - 6 o’clock position	7 - 9 o’clock position
Septal puncture	AP view	Pseudo AP view
	LAO view	RAO view
Crossing of mitral valve and balloon dilatation	RAO view	LAO view
